# Incorporating Transversus Abdominis Plane Block in Hysterectomy Anesthesia: Clinical Outcomes in a Hispanic Population

**DOI:** 10.7759/cureus.91339

**Published:** 2025-08-31

**Authors:** Francisco Lasanta Gorbea, Abner Limardo, Sabrina Valentin, Fernando Ojeda, Javier Torres, Fabian Ramirez-Rivera

**Affiliations:** 1 Department of Medical Education, St. Luke's Episcopal Hospital, Ponce, PRI; 2 Department of Ob/Gyn, St. Luke's Episcopal Hospital, Ponce, PRI; 3 Department of Anesthesiology, St. Luke's Episcopal Hospital, Ponce, PRI

**Keywords:** abdominal wall blocks, enhanced recovery after surgery, gynecologic surgery, minority health, opioid-sparing techniques, postoperative analgesia, regional anesthesia

## Abstract

Background

Effective postoperative pain management is essential to enhancing recovery after abdominal hysterectomy. Enhanced Recovery After Surgery (ERAS) protocols recommend opioid-sparing, multimodal analgesia, including regional techniques such as the transversus abdominis plane (TAP) block. Although evidence supports its use, data on its effectiveness in underrepresented populations remain limited. This study evaluated the impact of the TAP block on postoperative recovery in a Hispanic female population.

Methodology

We conducted a retrospective cohort study at St. Luke’s Episcopal Hospital, a tertiary medical center in Ponce, Puerto Rico. Medical records of 300 Hispanic women aged ≥21 years who underwent elective abdominal hysterectomy between August 2023 and September 2024 were reviewed. Patients were grouped based on receipt of a TAP block in addition to general anesthesia. Outcomes included time to ambulation, return of bowel function, hospital length of stay, time to first analgesic request, and incidence of constipation. Statistical analysis included t-tests and chi-square tests.

Results

Patients in the TAP block group demonstrated significantly better early ambulation, with 81 (98.8%) ambulating by postoperative day (POD) 1 compared to 197 (90.8%) in the control group. Bowel function recovery was similar on POD 1, 35 (42.7%) TAP vs. 101(46.3%) control, but significantly improved by POD 2 in the TAP group, 81 (98.8%) vs. 196 (89.9%) in the control group. Mean hospital stay was shorter in the TAP group (1.93 vs. 2.05 days). Time to first analgesic request was longer (115.5 vs. 88.6 minutes), and constipation incidence was lower 0 (0%) in the TAP group vs. 14 (6.4%), with a significant finding of *P *= 0.014.

Conclusions

TAP block use during abdominal hysterectomy improved recovery outcomes, reduced opioid-related side effects, and demonstrated benefit in an entirely Hispanic cohort. These findings support its integration into multimodal analgesia protocols for gynecologic surgery.

## Introduction

A hysterectomy, the surgical removal of the uterus, is one of the most performed gynecologic procedures worldwide, indicated for a range of conditions including abnormal uterine bleeding, fibroids, prolapse, and malignancy. Surgical approaches include abdominal, vaginal, laparoscopic, and robotic-assisted techniques, each with varying degrees of invasiveness, recovery times, and complication profiles. Although minimally invasive methods such as laparoscopic and robotic-assisted hysterectomies are increasingly favored due to their faster recovery and reduced morbidity, abdominal hysterectomy remains necessary in certain clinical scenarios, including large fibroids or extensive adhesions [1,2].

Effective postoperative pain control is essential to enhancing recovery, minimizing complications, and reducing opioid use. Enhanced Recovery After Surgery (ERAS) protocols recommend a multimodal, opioid-sparing approach to perioperative pain management. These strategies include the use of non-opioid analgesics and regional anesthetic techniques. Among these, the transversus abdominis plane (TAP) block has gained attention as a safe and effective regional technique that targets somatic innervation of the anterior abdominal wall (T10-L1). This layered approach helps minimize opioid consumption, reduce postoperative nausea and vomiting, and enhance patient satisfaction [3].

Although initially underutilized in gynecologic procedures, recent studies have demonstrated its safety and effectiveness in reducing early postoperative pain and opioid use, particularly in laparoscopic hysterectomies [4].

Evidence suggests that TAP blocks may improve early postoperative outcomes such as ambulation, bowel function recovery, and hospital discharge timing. However, prior studies have yielded inconsistent results, particularly regarding long-term pain relief, complication rates, and opioid-related side effects. Furthermore, most existing data is derived from non-diverse populations, limiting generalizability to underserved or minority groups.

Meta-analyses suggest that TAP blocks significantly reduce early postoperative pain scores, morphine requirements, and time to first analgesic use in hysterectomies performed for benign conditions. Preoperative administration appears more effective than postoperative use, supporting the concept of preemptive analgesia. While findings are encouraging, further large-scale studies with standardized reporting of functional and patient-centered outcomes are needed to fully validate these benefits. The lack of such comprehensive evidence has contributed to TAP blocks not yet being widely adopted as a standard practice [5].

This study aims to evaluate the clinical impact of TAP block implementation on postoperative recovery outcomes among an exclusively Hispanic female population undergoing a variety of hysterectomy types, including both open and minimally invasive approaches. By focusing on an underrepresented demographic and a diverse range of surgical techniques, this study contributes to a more inclusive and comprehensive understanding of multimodal analgesia strategies in gynecologic surgery.

## Materials and methods

We conducted a single-center, retrospective cohort study of patients who underwent elective hysterectomy at our institution between August 2023 and September 2024. Data were retrieved from the hospital’s electronic medical records system following authorization for access and de-identification. This study was approved by the Ponce Health Sciences University Institutional Review Board (IRB protocol number: 2410219765).

Electronic records were searched using International Classification of Diseases, Tenth Revision (ICD-10) codes for all types of hysterectomy procedures, including abdominal, laparoscopic, vaginal, and robotic-assisted approaches. Eligible patient records were randomly sampled using IBM SPSS Statistics to minimize selection bias and ensure a representative cohort. This process yielded a final sample of 300 patients who met the inclusion criteria. Inclusion criteria consisted of female patients aged 21 years or older who underwent elective abdominal, laparoscopic, vaginal, or robotic-assisted hysterectomy. Exclusion criteria included patients under 21 years of age, those undergoing multiple or concurrent surgical procedures, and those with incomplete medical records or unavailable data.

Data reviewed included demographic information, surgical approach, anesthesia techniques, and postoperative recovery outcomes. All data was entered into the institution’s REDCap system. Statistical analyses were conducted using IBM SPSS Statistics. Statistical methods included descriptive statistics, chi-square tests for categorical variables, linear regression analyses to assess relationships and influential factors between variables, and t-tests for comparing means between groups.

At our institution, the TAP block is part of the multimodal analgesia regimen and is administered preoperatively by the attending anesthesiologist. The procedure is performed under ultrasound guidance using 30 mL of 0.25% bupivacaine per side, distributed across three anatomical injection sites along each side of the abdominal wall to target the T10-L1 dermatomes. For this study, patients were classified based on whether they received a TAP block as part of their perioperative care.

## Results

In this study, 300 patients underwent elective hysterectomies. Two hundred and eighteen were assigned to the control group that received general anesthesia. Eighty-two patients were assigned to the exposed group that received a TAP block in addition to general anesthesia. Baseline demographics, including body mass index (BMI) and age, were comparable between the groups. The distribution among surgeries and exposure to TAP block is detailed in Table 1.

**Table 1:** Patient distribution by hysterectomy type

Hysterectomy type	Total of cases	General anesthesia (*n* = 218)	TAP block (*n* = 82)	*P*-value
Total laparoscopic	88 (29.3%)	68 (31.2%)	20 (24.4%)	0.249
Laparoscopic vaginal	10 (3.3%)	9 (4.1%)	1 (1.2%)	0.295
Total abdominal	151 (50.3%)	101 (46.3%)	50 (61%)	0.028
Subtotal abdominal	2 (0.7%)	1 (0.5%)	1 (1.2%)	0.473
Robotic-assisted, lap.	49 (16.3%)	39 (17.9%)	10 (1.2%)	0.311

Ambulation

The study measured on which postoperative day (POD) patients first ambulated. The TAP block group demonstrated higher ambulation rates by POD 1 (81, 98.8% vs. 197, 90.8%). Detailed results are shown in Table 2.

**Table 2:** First ambulation by POD with cumulative counts. Non-ambulatory patients were excluded from analysis (one per group). POD, postoperative day

POD	POD of first ambulation (% within group)	Cumulative count (running %)
General anesthesia (*n *= 217)		
POD 0	1 (0.5%)	2 (0.9%)
POD 1	195 (89.9%)	197 (90.8%)
POD 2	20 (9.2%)	217 (100.0%)
POD 3	1 (0.5%)	217 (100.0%)
TAP block (*n *= 81)		
POD 0	0 (0.0)	1 (1.2%)
POD 1	80 (98.8%)	81 (98.8%)
POD 2	1 (1.2%)	82 (100.0%)

Bowel function

The study also measured the return of bowel function (ROBF) after surgery in the patient cohort. ROBF was defined as the first occurrence of flatus after surgery. On POD 1, ROBF was elevated in the control group 101 (46.3%) versus 35 (42.7%) in the TAP group. By POD 2, ROBF was significantly higher in the TAP block group, 81 (98.8%) vs. 196 (89.9%) in the control group. All results are shown in Table 3.

**Table 3:** Return of bowel function (ROBF) by POD with cumulative counts. POD, postoperative day

POD	POD of ROBF (% within group)	Cumulative count (running %)
General anesthesia (*n *= 218)		
POD 0	1 (0.5%)	1 (0.5%)
POD 1	100 (45.9%)	101 (46.3%)
POD 2	95 (43.6%)	196 (89.9%)
POD 3	18 (8.3%)	214 (98.2%)
POD 4	3 (1.4%)	217 (99.5%)
POD 5	1 (0.5%)	218 (100%)
TAP block (*n* = 82)		
POD 0	0 (0.0%)	0 (0.0%)
POD 1	35 (42.7%)	35 (42.7%)
POD 2	46 (56.1%)	81 (98.8%)
POD 3	1 (1.2%)	82 (100%)
POD 4	0 (0.0%)	82 (100%)
POD 5	0 (0.0%)	82 (100%)

Postoperative complications

Both groups were followed for postoperative complications. There was a significant difference in the incidence of postoperative constipation in the general anesthesia group compared to the TAP block group (14, 6.4%, vs. 0, 0%; *P *= 014). Detailed results are demonstrated in Table 4.

**Table 4:** Postoperative complications and analgesics used during admission. NSAIDs, nonsteroidal anti-inflammatory drugs

Postop complications	Total of cases (*n* = 64)	General anesthesia (*n* = 218)	TAP block (*n* = 82)	*P*-value
Anemia	48 (75%)	38 (17.4%)	10	0.270
Hemorrhage	1 (1.6%)	1 (0.5%)	0 (0%)	0.539
Fluid overload	1 (1.6%)	1 (0.5%)	0 (0%)	0.539
Constipation	14 (21.9%)	14 (6.4%)	0 (0%)	0.014
Pain medications administered				
Morphine	289 (34.8%)	212 (37.7%)	77 (35.3%)	0.169
NSAIDs	274 (33.0%)	200 (35.6%)	74 (33.9%)	0.681
Acetaminophen	73 (8.8%)	45 (8.0%)	28 (12.8%)	0.015
Gabapentin	26 (3.1%)	15 (2.7%)	11 (5.0%)	0.06
Percocet	118 (14.2%)	90 (16.0%)	28 (12.8%)	0.259
Total	830 (100%)	562 (100%)	218 (100%)	

Length of stay

The mean hospital stay was shorter in the TAP group (1.93 days) compared to the control group (2.05 days), though this difference was not significant (*P *= 0.062). Details given in Table 5.

**Table 5:** Hospital length of stay by group.

	*t*-test mean analysis		*P*-value
	General anesthesia (SD)	TAP block (SD)	
Length of stay (days)	2.05 (1.71) (*n* = 218)	1.93 (0.94) (*n* = 82)	0.062
Time to first analgesic request by the patient (minutes)	89 (139.7) (*n* = 156)	116 (120.9) (*n* = 63)	0.646

## Discussion

In our study, adding a TAP block to standard general anesthesia significantly improved early postoperative outcomes, including ambulation and bowel function recovery, in elective abdominal hysterectomy patients. The TAP block group demonstrated higher ambulation rates by POD 1 (81, 98.8%, vs. 197, 90.8%) and earlier return of bowel function on POD 2 (81, 98.8%, vs. 196, 89.9%). The outcomes are aligned with the goals of ERAS protocols, which advocate for multimodal, opioid-sparing strategies to enhance early recovery and patient satisfaction [6,7].

A key finding in our study was the absence 0 (0%) of postoperative constipation in the TAP group, compared to an incidence of 14 (6.4%) in the control group (*P* = 0.014). This absence of constipation is a significant clinical finding that contributes evidence to the benefits of an opioid-sparing regimen. All these findings are consistent with the existing literature, suggesting that multimodal, opioid-sparing approaches improve patient recovery [8,9].

While there was no statistically significant difference in hospital length of stay between the TAP block and control groups (1.93 vs. 2.05 days, respectively) (*P* = 0.062), the mean difference reported in our study is consistent with trends noted in previous studies. While the length of stay is a multifactorial variable, such as institutional discharge protocols, the clinical relevance of a shorter hospital stay cannot be dismissed. Shorter stays in the hospital translate to significant cost savings and reduced healthcare system burdens. Future studies incorporating cost-effectiveness analyses would be valuable to assess whether the benefits of TAP blocks outweigh the added procedural expense.

Our findings were consistent with Bacal et al. [5], in which our TAP block group also had a delayed need for first analgesic administration. Some earlier studies, like Shin et al., have found limited benefits outside the post-anesthesia care unit or no significant impact on length of hospital stay [10]. Our findings expand on this by demonstrating measurable benefits in recovery of bowel function and ambulation, suggesting that TAP blocks can be useful outside the immediate postoperative setting. However, it is important to note that Shin et al. used only minimally invasive surgeries as part of their study, which may differ from the outcomes observed in our study, which included multiple modes of hysterectomy [10]. Including patients who underwent open abdominal hysterectomy provides a broader context for evaluating the effectiveness of TAP blocks across different surgical approaches.

Although our study design was non-randomized and retrospective, the trends observed in our cohort support the growing body of evidence advocating for incorporating TAP blocks into routine pain management protocols. Further prospective randomized trials, with a focus on long-term follow-up, opioid use, and patient satisfaction, are needed to strengthen these findings and make TAP blocks a standard of care for abdominal surgery, open and minimally invasive.

While our study provides valuable insights into the impact of the TAP block on postoperative recovery in a Hispanic population, several limitations must be acknowledged. First, this was a retrospective, single-center study, which limits the generalizability of the findings to other institutions or populations. Second, the lack of randomization introduces potential selection bias, as the groups may differ in unmeasured ways that could influence outcomes. Additionally, our analysis did not account for the potential variability in anesthesia techniques or the influence of different hysterectomy types on recovery outcomes. Finally, long-term outcomes beyond the immediate postoperative period were not assessed, which would be important for evaluating the full benefits of TAP blocks over time.

## Conclusions

Our findings support the integration of TAP blocks into multimodal perioperative pain management for abdominal hysterectomy, in alignment with current ERAS protocols. Given the positive trends in early recovery metrics, TAP blocks may offer broader benefits across abdominal surgeries. Future studies should determine the place of TAP blocks in achieving maximal recovery, improving patient satisfaction, and cost-effective outcomes in abdominal surgery.
